# Cytokine changes in nasal secretions after acute acoustic trauma

**DOI:** 10.5937/jomb0-54742

**Published:** 2025-06-13

**Authors:** Vanja Jovanović, Sonja Marjanović, Aleksandar Perić, Dejan Jovanović, Zoran Radojičić, Mirjana Đukić, Danilo Vojvodić

**Affiliations:** 1 University of Defence, Military Medical Academy, Faculty of Medicine, Belgrade; 2 Military Medical Academy, Institute of Occupational Health, Belgrade; 3 Military Medical Academy, Department of Otorhinolaryngology, Belgrade; 4 Military Medical Academy, Institute of Radiology, Belgrade; 5 University of Belgrade, Faculty of Organizational Sciences, Belgrade; 6 University of Belgrade, Faculty of Pharmacy, Department of Toxicology, Belgrade; 7 Military Medical Academy, Institute for Medical Research, Division of Clinical and Experimental Immunology, Belgrade

**Keywords:** audiometry, cytokines, gunfire, gender, hearing loss, impulse noise, interleukin-1b, nasal cavity, audimetrija, citokini, gubitak sluha, impulsna buka, interleukin-1b, nosna šupljina, pol, pucnjava

## Abstract

**Background:**

Occupational noise-induced hearing loss poses a significant health risk, especially for military and law enforcement. Unlike steady, prolonged noise, impulse noise, such as gunfire, leads to more immediate and severe hearing impairment. This study explores the link between sensorineural hearing loss and cytokine levels in nasal secretions of male and female military cadets exposed to high-intensity gunfire noise.

**Methods:**

105 cadets (75 males and 30 females, aged 19-25) undergoing regular firearms training were included. Audiometric testing and measurement of IL-1b, TNF-a, GM-CSF, and IL-10 concentrations in nasal secretions were conducted at three time points: 24 hours before, immediately after and 24 hours following noise exposure.

**Results:**

Acute acoustic trauma affected the hearing of 39% of male and 21% of female participants. Among those with audiometric changes, IL-1b levels increased immediately after exposure, while both IL-1b and GM-CSF levels were elevated 24 hours later. Nasal IL-1b and GM-CSF levels significantly changed across all participants, with the most pronounced alterations seen in those with audiogram changes.

**Conclusions:**

Acoustic trauma associated with a certain degree of hearing loss due to noise exposure is linked to an increase in nasal pro-inflammatory cytokine levels, and this effect is more pronounced in men than in women.

## Abstract (sr)

**Uvod:**

Profesionalno oštećenje sluha izazvano bukom predstavlja značajan zdravstveni rizik, posebno za vojna i policijska lica. Za razliku od konstantne, dugotrajne buke, impulsna buka, poput pucnjave, uzrokuje brža i ozbiljnija oštećenja sluha. Ova studija istražuje povezanost senzorineuralnog gubitka sluha i nivoa citokina u nosnom sekretu kod vojnih kadeta oba pola, izloženih visokointenzivnoj buci tokom pucanja.

**Metode:**

105 kadeta (75 mu{karaca i 30 žena, starosti 19-25 godina) je učestvovalo u ovom istraživanju. Audiometrijsko testiranje i merenje koncentracija IL-1b, TNF-a, GM-CSF i IL-10 u nazalnim sekretima sprovedeni su u tri vremenska intervala: 24 sata pre, odmah nakon, i 24 sata posle izloženosti buci.

**Rezultati:**

Akustična trauma izazvana bukom uticala je na sluh kod 39% muških i 21% ženskih učesnika. Kod onih sa promenama na audiogramu, nivo IL-1b porastao je odmah nakon izloženosti, dok su nivoi IL-1b i GM-CSF bili povećani 24 sata kasnije. Nivoi nazalnih IL-1b i GM-CSF značajno su se promenili kod svih učesnika, sa najizraženijim promenama kod onih sa promenjenim audiogramima. Muški kadeti su pokazali više vrednosti odnosa proupalnih/anti-upalnih citokina u poređenju sa ženskim kadetima.

**Zaključak:**

Akustična trauma povezana sa određenim stepenom gubitka sluha usled izlaganja buci pucnja udružena je sa povećanjem nivoa nazalnih proinflama tornih citokina i izraženija je kod muškaraca u odnosu na žene.

## Introduction

Noise can harm health depending on its intensity, frequency, and duration [1]. By itself, noise and sound, often used interchangeably, refer to different concepts: sound is a physical phenomenon (vibrations that travel through air or another medium), while noise is a subjective interpretation of specifically unwanted sound that disrupts or causes discomfort [2]. Sound’s elements, such as frequency (the number of sound wave oscillations per second, unit: Hz), intensity (sound strength or loudness, unit: dB), pitch (the tone’s highness or lowness based on frequency), and amplitude (which affects loudness based on wave height) are contributing factors to hearing impairment, each influencing it in a distinct manner [3]. According to the American National Institute of Health (NIH), noise exposure above 50 dB on public health in the workplace or environment is of particular concern, including hearing impairment and stress-associated diseases, including cardiovascular disease and diabetes [4] [5].

Noise-induced hearing loss (NIHL) is a significant health concern, particularly in high-risk occupational settings such as construction, manufacturing, transportation, and law enforcement [6] [7]. Approximately 5% of the global population suffers from NIHL, with a higher prevalence observed in adult males [8] [9] [10] [11] [12] [13].

In contrast to the steady, prolonged exposure of construction and manufacturing workers to high-intensity noise (85–110 dB, often in the 2,000-4,000 Hz frequency range), military and law enforcement personnel are exposed to impulse noise, sudden and extremely loud noises such as gunfire (140–180 dB, predominantly in the 3,000–4,000 Hz frequency range) [14] [15].

Gunfire noise ranks as a fourth-degree noise [12] [16]. The distinct types of exposure, steady and prolonged versus sudden and intense, result in different patterns of hearing impairment, with the latter often leading to immediate and severe auditory damage [17]. Noise exposure to frequencies in the 3,000–4,000 Hz range, common in both occupational and impulse noise environments, can lead to acute acoustic trauma, either causing a temporary threshold shift (TTS) or permanent threshold shift (PTS) depending on the intensity and duration of exposure, with TTS typically resolving within 24–48 hours [10] [18] [19] [20] [21].

Driving NIHL encompass mechanical damage in the inner ear, inflammation, oxidative stress, reduced blood flow, and excessive stimulation of sensory cells and nerve endings in the cochlea [14] [22] [23] [24]. The similarities between the nasal and middle ear mucosa support the idea that nasal secretions can reflect immune responses in the middle and inner ear. Studies (animal and human) indicate that inflammation in the middle ear can trigger coordinated immune responses in both regions, and inflammatory mediators may diffuse through the oval and round window into the inner ear, including the cochlea [25] [26] [27]. Given the immune similarities between nasal and middle ear mucosa, cytokine levels in nasal secretions may reflect inflammation in related ear structures [27].

Animal studies have shown immune coordination between the middle and inner ear during inflammation, with cytokine levels in nasal and middle ear secretions correlated, suggesting shared immune mechanisms [25] [26] [28] [29].

Based on the available literature, we hypothesised that high-intensity noise exposure activates immune responses linking the inner ear and nasal mucosa, with potential gender differences in this immune response [30] [31]. To test this hypothesis, we measured nasal secretions of pro-inflammatory cytokines – IL-1β (linked to acute inflammatory responses), TNF-α (key in systemic inflammation and acute-phase reactions), and GM-CSF (which stimulates immune cell production and inflammation in response to injury) – as well as the anti-inflammatory cytokine IL-10 (which regulates immune responses and limits excessive inflammation). Measurements were taken from male and female cadets before and after regular shooting exercises, and the changes were correlated with audiometrically measured hearing impairment.

## Materials and methods

### Participants, inclusion, and exclusion criteria

This study involved 105 cadets (healthy cadets of both sexes, aged 19 to 25 years, all Caucasian), an extensive sample for this type of research, considering the special conditions, including military personnel. Despite these challenges, we recruited the maximum number of participants allowed, comparable to similar studies such as Guida et al. [20]. Audiometric tests and nasal secretion samples were taken at three time points: 24 hours before, immediately after and 24 hours after regular shooting practice with the CZ 99 pistol, during which protective measures (earplugs) were used. The use of antiphons during shooting is prescribed by the applicable regulations only in closed shooting ranges. This is when PELTOR speakers are used (with or without a Bluetooth communication system). In field conditions, on open or semi-open ranges (which we used), earplugs are not mandatory and are not included in the equipment kit for this gun. For ethical reasons, our subjects used 3M E-A-R Soft Neon earplugs with adhesive tape. The results refer to shooting under the same conditions for all subjects: same target, same number of shots, same distance, and same shooting range. Each subject fired 15 shots at the target from a distance of 20 meters.

The shooting took place in an open area, the socalled semi-enclosed shooting range (the subjects were under the roof, which was their shooting environment). Exclusion criteria for the study were acute and chronic diseases of the respiratory tract and other organs, including inflammatory, infectious and allergic diseases of the ear, nose and throat, and lateralisation in the audiological findings. In addition, participants with acute illnesses or injuries in the previous 7 days and noise exposure in the previous 24 hours were excluded. The same applied to injuries of any kind. Only healthy cadets took part in the study. The criteria for the coveted cadets were to be healthy and to take part in the obligatory shooting exercises with a CZ99 pistol that was part of their training. Before and after the shooting exercises, all participants underwent regular, systematic examinations for military service, including an otorhinolaryngologic examination, which confirmed that they were completely healthy. The participants did not wear facemasks; we wanted to respect all the realistic conditions that prevailed during military training. CZ99 pistols were used, which left very little gunpowder residue. The cadets shot while standing, in a posture with outstretched arms, so that gunpowder particles remain on the skin of the fingers and hands and do not reach the face. As this is a modern weapon, there was no smoke.

Tympanometry was performed before and after the tests to rule out participants with conductive hearing loss (inflammation, otosclerosis, tympanosclerosis, etc.) and to detect possible damage to the middle or inner ear during noise exposure.

### Audiometric test

Participants underwent pure tone audiometry (PTA) to determine hearing thresholds at three intervals: 24 hours before (baseline), immediately after and 24 hours after exposure to gunshot noise. The tests were conducted in a soundproof booth complying with ANSI and ISO standards using a calibrated Bell »Plus« audiometer (Inventis, Italy). Pure tone thresholds (PTTs) were measured for air conduction (AC) at frequencies of 125–8000 Hz and for bone conduction (BC) at 250–4000 Hz, with masking applied where necessary.

The average PTT values for AC and BC were calculated according to the guidelines of the American Academy of Otolaryngology-Head and Neck Surgery (AAO-HNS) Committee on Hearing and Equilibrium [32]. Sensorineural hearing loss was defined as a continuous decline in air and bone conduction thresholds without an air-bone gap. No air-bone gaps were detected in the study participants [33]. A continuous decline in the curve of air and bone conduction thresholds without an air-bone gap indicated hearing loss in the participants. The audiometer settings included intensities up to 120 dB HL for AC and 80 dB HL for BC; frequency range 125–8000 Hz (125 kHz available with HDA-280 headphones); Input tone, variable tone; Standards: EN 60645- 1/ ANSI S3.6, Type 3.

### Nasal secretion sampling and flow cytometric analysis

Nasal secretion samples were collected from participants using cotton swabs (10 mm x 4 mm) inserted into the middle nasal meatus for 5 minutes until saturation. The swabs were then placed in Eppendorf tubes with 1 mL of transfer medium for 30 minutes to allow diffusion of mediators. Following centrifugation, supernatants were frozen at -70°C for later analysis. Cytokine concentrations (IL-1β, TNF-α, IL-10, and GM-CSF) were measured using flow cytometry kits (YSL multiplex proteomic cytokine kits, R & D Systems, Inc) on a Beckman Coulter Navios flow cytometer, with results expressed in pg/mL. According to the manufacturer, the flow cytometry method’s intra- and inter-assay CVs are <10%, ensuring reliability and reproducibility.

### Limitations of the study

Possible weaknesses that could affect the interpretation or generalizability of the results are: I) study conditions: the study was conducted in a field and not in a controlled laboratory setting. Due to the limited space on the shooting range, only about 35 cadets could participate at any one time. Environmental influences such as occasional rain, wind, or slight fluctuations in humidity and temperature were mitigated by conducting the study on several consecutive Saturdays to minimise their effects; II) methodology: sampling and audiometry at the time point II were performed immediately after shooting practice in a silent room of the shooting range facility, as opposed to the time points I and III, which took place in a laboratory; and III) ethical constraints: for safety reasons, participants could not be exposed to higher noise levels from larger weapons without strong hearing protection, which is mandatory in such cases.

### Ethics

The study was conducted at the Military Academy and the Military Medical Academy (Institute of Occupational Medicine, Institute of Medical Research) within the framework of the research project MFVMA 01/20-22, from November 2022 to April 2023. Approval for this study was obtained from the Ethics Committee of the Faculty of Medicine of the Military Medical Academy. All participants signed informed consent. The study was conducted as an interventional prospective study.

### Statistical analysis

SPSS version 26.0 software (IBM, USA, 2019) was used for data analysis. Continuous variables are presented as mean ± standard deviation or median with interquartile range (25th and 75th percentiles), while categorical variables are presented as frequencies of the individual categories. The Mann-Whitney U test was used for comparison between groups, and the Wilcoxon test was used for pairwise comparison within a group. We used ANOVA for the parametric distribution of continuous variables that were compared within groups at three different time points. A value of p<0.05 is considered significant in all analyses. Results are presented as mean ± standard deviation (SD).

## Results

The levels of the nasal pro-inflammatory cytokines IL-1β, TNF-α, GM-CSF, and the anti-inflammatory cytokine IL-10 in participants with and without hearing impairment are presented in tables. Nasal cytokine levels 24 hours before shooting (pre-shooting, time point I) were used as control values, with each participant serving as their own control. The post-shooting values, immediately after shooting (time point II) and 24 hours later (time point III), were used to categorise participants into groups with and without acute hearing impairment after shooting (groups: YES, NO, 10 dB, and 5 dB). Gender differences were also accounted for in the analysis and statistical processing of the results.

**Figure 1:**
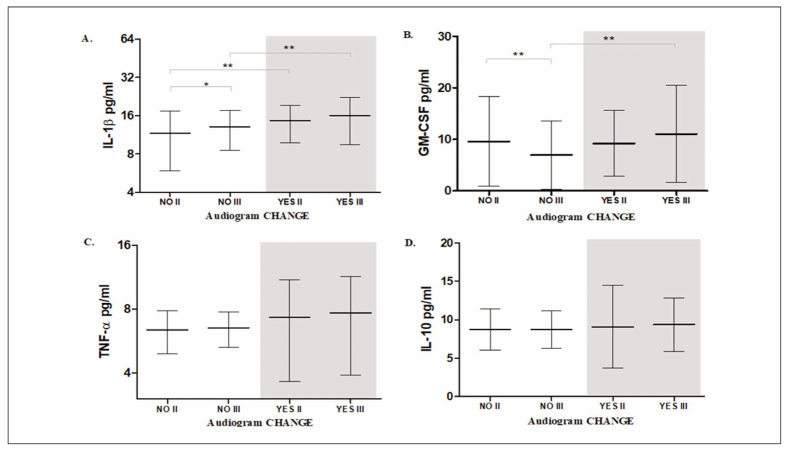
Cytokine concentrations in groups with and without audiogram changes after the shooting. Results are expressed in pg/mL and presented as mean ± standard deviation (SD). Levels of statistical significance: * - p<0.05; ** -p<0.01

Nasal IL-1β and GM-CSF levels increased following shooting in participants with altered audiograms. Nasal IL-1β rose significantly imme diately after shooting (time point II) and remained elevated (time point III), along with elevated nasal GM-CSF secretion in participants with altered audiograms (YES group), compared to those without hearing changes (NO group) (Table 1, Table 2, Figure 1).

**Table 1:** Analysis of nasal cytokine concentrations between groups with or without audiogram changes after shooting (Mann-Whitney test).

Time point	Groupn=105	IL-1β	TNF-α	GM-CSF	IL-10
II	10 dB	12.16 ± 3.01 ^h^	8.55±5.87	11.27±5.75	7.65±1.37
	5 dB	15.36±5.52 ^d,h^	6.27±2.16	8.74±6.54	9.58±6.52
	YES a.c.	14.26±4.99 ^a^	7.03±3.85	9.24±6.34	8.91±5.36
	NO a.c.	11.94±6.18 ^a,d^	6.53±1.78	9.46±8.70	8.72±2.67
III	10 dB	14.84±4.05	6.42±1.53	12.57±5.91 ^f^	7.70±1.25
	5 dB	16.53±7.40 ^e^	8.14±4.43	10.27±10.79 ^g^	10.06±4.05
	YES a.c.	15.97±4.53 ^b^	7.64±3.74	10.65±9.46 ^c^	9.36±3.50
	NO a.c.	13.08±6.24 ^b,e^	6.51±1.24	6.96±6.59 ^c,f,g^	8.69±2.45

**Table 2:** YES a.c. – all subjects with audiogram change; NO a.c. – subjects without audiogram change; 10 dB – subjects with 10 dB audiogram reduction; 5 dB – subjects with 5 dB audiogram reduction; audiometry and nasal secretion samples were performed at three time points: I – 24 hours before, II – immediately after, and III – 24 hours after gunshot exposure (a regular shooting exercise with the CZ 99 pistol using earplugs); n=105 (military cadets of both sexes, healthy, all Caucasian, aged 19–25 years). Cytokine levels are expressed in pg/mL and given as mean ± standard deviation. Levels of statistical significance: * – p0.05; ** – p0.01

^a^ IL-1β	group All dB	II	>	group 0 dB	II	** MW
^b^ IL-1β	group All dB	III	>	group 0 dB	III	** MW
^c^ GM-CSF	group All dB	III	>	group 0 dB	III	* MW
^d^ IL-1β	group 5 dB	II	>	group 0 dB	II	** MW
^e^ IL-1β	group 5 dB	III	>	group 0 dB	III	** MW
^f^ GM-CSF	group 10 dB	III	>	group 0 dB	III	* MW
^g^ GM-CSF	group 5 dB	III	>	group 0 dB	III	* MW
^h^ IL-1β	group 5 dB	II	>	group 10 dB	II	* MW

The degree of hearing loss correlated with variations in cytokine levels. Specifically, higher IL-1β concentrations were observed in the 5 dB hearing loss group compared to the 10 dB group (time points II and III) (Table 1).

Acute noise trauma alters nasal IL-1β and GM-CSF secretion in participants without hearing reduction. Values of the nasal IL-1β levels are significantly higher at time point III in the NO group (p<0.05) and slightly higher in the YES group, compared to time point II (time points: III>II, Mann-Whitney U test). A significant decrease in nasal IL-1β levels was observed at time point II, followed by a significant increase at time point III (time points I>II< II, Wilcoxon test). Regarding nasal GM-CSF secretion, the YES group showed a similar trend to IL-1β, with a slight, non-significant increase from time point II to III, while the NO group exhibited a significant decrease between these time points (p<0.01).

Nasal TNF-α level was slightly elevated in the YES group, with no significant differences observed between time points II and III.

Nasal IL-10 levels remained consistent across YES and NO groups and time points II and III. Accordingly, the ratio of IL-1β/IL-10 (pro-inflammatory to anti-inflammatory cytokines) depends on changes in nasal IL-1β. This ratio increased in the NO group at time point III compared to time point II, as confirmed by the Wilcoxon and Mann-Whitney U tests. A comparison of the mean IL-1β/IL-10 ratio between the YES and NO groups revealed a significant increase in the YES group before the shooting (time point I, YES>NO).

Acute noise trauma affects men more often than women. Altered audiograms were observed in 34% of all participants (36/105), with a significantly higher incidence in men than women (39% vs. 21%, *p*=0.0055, chi-square test). However, all participants with altered audiograms experienced sensorineural hearing loss at 4000 Hz.

Gender differences were also noted in participants with and without audiogram changes. Both women and men from the YES group had lower IL-10 before the shooting and, the opposite, higher IL-10 values after the shooting (time point III), indicating the association between the hearing reduction and secretion of pro-inflammatory IL-10 cytokine. Nasal secretion of pro-inflammatory cytokines GM-CSF and IL-1β follows a similar pattern: both are lower in the NO groups compared to the YES groups in both men and women across all three time points; a significant difference is observed at time point III in women (Table 3, Table 4).

**Table 3:** Analysis of the difference in average nasal cytokine concentration within female and male groups (Mann-Whitney test).

Time point	Group<br>n=105	IL-1β	TNF-α	GM-CSF	IL-10
** I **	**Females YES**<br>**Females NO**	14.26±4.20<br>12.03±5.15	6.39±0.70<br>6.69±1.19	9.87±6.35<br>8.30±6.48	**8.05±0.36 ^d ^** <br>9.21±2.89
	**Males YES**<br>**Males NO **	**14.92±6.74 ^a ^** <br> **14.20±7.07 ^a^**	6.95±1.50<br>6.95±2.12	10.45±6.61<br>9.35±6.34	**8.96±1.61 ^d,e ^** <br>9.21±2.92
** II **	**Females YES**<br>**Females NO**	15.56±8.04<br>11.14±6.60	6.86±0.98<br>8.21±6.32	10.42±7.62<br> **9.92±6.07 ^c^**	8.12±4.49<br>9.83±3.12
	**Males YES**<br>**Males NO **	13.44±3.76<br>11.74±4.51	6.45±1.41<br>6.47±1.59	9.39±6.15<br>7.95±5.84	**8.05±2.25 ^e^** <br>8.44±2.64
** III **	**Females YES**<br>**Females NO**	**17.84±7.25 ^b ^** <br> **12.30±5.25 ^b^**	7.11±1.54<br>6.41±1.41	10.27±6.39<br> **6.35±5.23 ^c^**	9.60±3.29<br>8.50±2.08
	**Males YES**<br>**Males NO **	14.96±5.52<br>13.52±4.33	7.46±3.57<br>6.52±1.18	8.64±6.32<br>8.26±7.70	9.36±3.13<br>8.49±2.25

**Table 4:** Audiometry and nasal secretion samples were taken at three time points: I – 24 hours before, II – immediately after, and III – 24 hours after gunshot exposure (a regular shooting exercise with CZ 99 pistol using earplugs); n=105 (military cadets of both sexes, healthy, all Caucasian, aged 19–25 years). Cytokine levels are expressed in pg/mL and given as mean ± standard deviation. Levels of statistical significance: * – p0.05

**^a^ IL-1β **	I time point	** Males YES **	>	** Males NO **	*
**^b^ IL-1β **	III time point	** Females YES **	>	** Females NO **	*
**^c^ GM-CSF **	II / III time point	** Females NO **	>	** Females NO **	*
**^d^ IL-10 **	I time point	** Males YES **	>	** Females YES **	*
**^e^ IL-10 **	I / II time point	** Males YES **	>	** Males YES **	*

The ratio IL-1β/IL-10 showed a higher value in time point III in female participants from NO groups and in time point I in the females from the YES group. The comparison of women and men showed an increase in the IL-1β/IL-10 and TNF-α/IL-10 ratio in the time point II in the NO group.

A small portion of the statistical data for IL-10, as shown in Table 5 (not the complete analysis for IL-10), illustrates the methodology and results due to the abundance of data. Confidence intervals for other cytokines were not presented due to the extensive data.

**Table 5:** Confidence intervals for il-10 across different groups and conditions. M – male cadets; F – female cadets; 10 dB – subjects with 10 dB audiogram reduction; 0 dB – subjects with 0 dB audiogram reduction; audiometry and nasal secretion samples were taken at three time points: I – 24 hours before, II – immediately after, and III – 24 hours after gunshot exposure (a regular shooting exercise with the CZ 99 pistol using earplugs); n=105 (military cadets of both sexes, healthy, all Caucasian, aged 19–25 years). Lower and upper 95% CI: the lower and upper limits of the 95% confidence interval, indicating the lowest and highest values, respectively, within which the true mean is expected to lie 95% of the time.

IL-10 Group (time points)	Group n=105	0 dB (M)	10 dB (M)	0 dB (F)	10 dB (F)
** I **	Lower 95% CI	0.7763	0.7693	0.6091	-1.257
	Upper 95% CI	1.345	1.725	1.193	5.920
** II **	Lower 95% CI	0.5294	0.8408	0.7108	-0.5242
	Upper 95% CI	1.816	1.303	1.221	3.791
** III **	Lower 95% CI	0.7287	0.6501	0.4907	0.3637
	Upper 95% CI	1.100	1.465	0.9197	1.796

## Discussion

**Figure 2:**
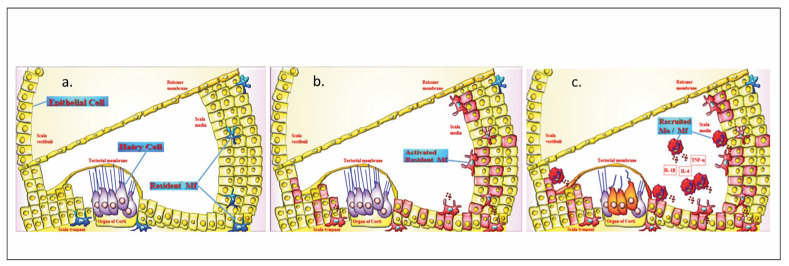
Changes in the inner ear under the influence of impulse noises. Figure 2 capture. a. Normal structure of the inner ear; b. Changes in the inner ear in cadets without audiogram change; c. Changes in the inner ear in cadets with audiogram change.

Due to its blood-labyrinth barrier, the inner ear has long been regarded as an »immune-privileged« organ. Still, recent studies have shifted this view by identifying the cochlea’s inflammatory response as a key factor in NIHL [34] [35]. Acoustic trauma, such as exposure to gunfire noise, induces significant immunological responses in the nasal secretions, which vary by gender and degree of hearing impairment; also, it can cause significant changes in the inner ear (Figure 2) [32] [36] [37] [38] [39] [40] [41] [42].

Our study found a higher incidence of hearing loss in men (39%) compared to women (21%) (*p*=0.0055), consistent with previous research indicating gender-specific differences in auditory sensitivity and resilience to noise damage, and factors related to brain development, behaviour, and neuromodulation [43] [44]. Men are more susceptible to acoustic trauma, potentially due to testosterone’s role in enhancing pro-inflammatory responses and suppressing anti-inflammatory pathways in the cochlea, as observed by Arslan et al. [45] [46] [47]. However, studies with predominantly male participants, such as those on firearm-related acoustic trauma in Finland and a Brazilian study of police officers, limit the ability to assess gender differences in NIHL susceptibility [20] [45].

Our results suggest that varying levels of hearing loss correlate with distinct cytokine concentrations in nasal secretions, a relationship illuminated by recent insights into the lymphatic connections between the upper respiratory tract and inner ear.

Studies in animal models show that antigen-sensitised cells recruited to the endolymphatic sac may originate from the lymphoid tissue of the nose and throat, suggesting an immunological link between the upper airways and the inner ear, where immune responses to nasal antigens may influence inner ear immunity and susceptibility to hearing loss following noise exposure [48] [49]. An immunoprotective role and the importance of the endolymphatic sac, an organ of the auditory and vestibular system, was confirmed by Kämpfe Nordström et al. [50]. Within this sac, macrophages interact with other immune cells, display migratory behaviour, and express immune markers, indicating a dynamic role in both innate and adaptive immunity for the inner ear [40] [51]. The endolymphatic sac holds a full complement of immunocompetent cells and functions as a critical immune controller for the inner ear, akin to organs in the mucosa-associated lymphoid tissue (MALT) system, which coordinates immune memory responses across different mucosal sites [52]. Clinical studies have shown a positive correlation between cytokines and chemokines in nasal and middle ear secretions, suggesting a shared immune response and similar production of Th1/Th2/Th17 inflammatory mediators in these regions [25]. Also, clinical evidence links chronic rhinosinusitis with sensorineural hearing loss, indicating that inflammation in the nasal and sinus mucosa can influence the inner ear’s immune processes, potentially leading to hearing impairment [27] [53].

We observed significant increases in nasal IL-1β and GM-CSF levels following noise exposure, particularly in participants with hearing loss. These cytokines are integral to the inflammatory response, and their elevation suggests that noise exposure triggers a cascade of immune activation. IL-1β, a pro-inflammatory cytokine, spiked immediately after shooting and remained elevated for 24 hours, while GM-CSF levels increased with the severity of hearing loss. These changes are consistent with cytokine production in the nasal mucosa, reflecting the acoustic trauma’s extent [54] [55] [56].

Elevated IL-1β levels in participants with a 5 dB hearing loss compared to those with a 10 dB loss indicate a complex inflammatory response that may not directly correlate with hearing loss severity. This supports previous findings that inflammation can support tissue repair and contribute to hearing loss [54] [55] [57].

Gender differences were also evident in cytokine patterns. Men exhibited a higher IL-1β/IL-10 ratio, indicating a stronger pro-inflammatory response than women, who showed higher IL-10 levels in the NO group. This suggests that hormonal influences, particularly androgens, modulate immune responses, contributing to gender-linked differences in NIHL susceptibility [31] [46] [58] [59]. GM-CSF, typically associated with protective roles in animal models, was elevated in individuals with more severe hearing loss, hinting at a more complex immune response in humans that may not be entirely protective.

Our analysis also revealed insights into other immune markers, such as TNF-α and IL-10. TNF-α levels did not significantly change after noise exposure, contrasting with animal models where TNF-α plays a role in cochlear inflammation [57] [60] [61]. IL-10, an anti-inflammatory cytokine, showed minimal changes across time points, except in women, where it was elevated post-exposure. This highlights a potential gender difference in the regulation of inflammatory responses. The increased IL-1β/IL-10 and TNF-α/IL-10 ratios in men point to a more pronounced inflammatory state, which may explain their greater susceptibility to NIHL. Testosterone may promote this pro-inflammatory response by enhancing IL-1β production and suppressing TNF-α in cochlear regions, as suggested by previous studies on hormonal influences on inflammation [46] [59].

## Conclusion

Our study demonstrates that acute exposure to gunfire noise induces differential cytokine responses in the nasal mucosa. Men exhibit a stronger proinflammatory profile associated with a higher incidence of hearing impairment. Elevated IL-1β and GM-CSF levels post-exposure correlate with hearing loss severity, suggesting that nasal cytokine measurements may be biomarkers for acoustic trauma risk. These findings highlight the need for further research to understand how immune responses in the upper airway may influence susceptibility to noise-induced hearing damage. The observed gender-specific differences in cytokine dynamics emphasise the need to consider biological sex in prevention strategies against NIHL. Controlled experimental studies could clarify the specific role of pro-inflammatory cytokines IL-1β, GM-CSF, and TNF-α vs. anti-inflammatory cytokine IL-10 in the context of noise trauma and immune resilience.

## Dodatak

### Acknowledgements

This study was supported by a Grant from the Ministry of Defence of the Republic of Serbia (Project No. MFVMA 01/20-22), and the Ministry of Science funded this research, Technological Development and Innovation, the Republic of Serbia, through a grant agreement with the University of Belgrade – Faculty of Pharmacy No: 451-03-47/2023-01/200161.

### Conflict of interest statement

All the authors declare that they have no conflict of interest in this work.

### List of abbreviations

IL-1β, interleukin-1 beta;<br>TNF-α, tumour necrosis factor alfa;<br>GM-CSF, granulocytemacrophage colony-stimulating factor;<br>IL-10, interleukin 10.
